# The Colonic Crypt: Cellular Dynamics and Signaling Pathways in Homeostasis and Cancer

**DOI:** 10.3390/cells14181428

**Published:** 2025-09-11

**Authors:** Anh L. Nguyen, Molly A. Lausten, Bruce M. Boman

**Affiliations:** 1 Cawley Center for Translational Cancer Research, Helen F. Graham Cancer Center & Research Institute, 4701 Ogletown-Stanton Road, Newark, DE 19713, USA; nguyena@udel.edu (A.L.N.); mlausten@udel.edu (M.A.L.); 2 Department of Biological Sciences, University of Delaware, Newark, DE 19716, USA; 3 Department of Cell & Developmental Biology, Thomas Jefferson University, Philadelphia, PA 19144, USA; 4 Department of Pharmacology & Experimental Therapeutics, Thomas Jefferson University, Philadelphia, PA 19144, USA

**Keywords:** colonic crypt, stem cells, goblet cells, tuft cells, M cells, enteroendocrine cells, Paneth-like cells, signaling pathways, colorectal cancer, epithelial–mesenchymal transition

## Abstract

The goal of this review is to expand our understanding of how the cellular organization of the normal colonic crypt is maintained and elucidate how this intricate architecture is disrupted during tumorigenesis. Additionally, it will focus on implications for new therapeutic strategies targeting Epithelial–Mesenchymal Transition (EMT). The colonic crypt is a highly structured epithelial unit that functions in maintaining homeostasis through a complex physiological function of diverse cell types: SCs, transit-amplifying (TA) progenitors, goblet cells, absorptive colonocytes, Paneth-like cells, M cells, tuft cells, and enteroendocrine cells. These cellular subpopulations are spatially organized and regulated by multiple crucial signaling pathways, including WNT, Notch, Bone Morphogenetic Protein (BMP), and Fibroblast Growth Factor (FGF). Specifically, we discuss how these regulatory networks control the precise locations and functions of crypt cell types that are necessary to achieve cellular organization and homeostasis in the normal colon crypt. In addition, we detail how the crypt’s hierarchical structure is profoundly perturbed in colorectal cancer (CRC) development. Tumorigenesis appears to be driven by LGR5+ cancer stem cells (CSCs) and the hyperproliferation of TA cells as colonocytes undergo metabolic reprogramming. Goblet cells lose their secretory phenotype, while REG4+ Paneth-like cells foster SC niches. Tumor microenvironment is also disrupted by upregulation of M cells and by tumor-immune crosstalk that is promoted by tuft cell expansion. Moreover, the presence of enteroendocrine cells in CRC has been implicated in treatment resistance due to its contribution to tumor heterogeneity. These cellular changes are caused by the disruption of homeostasis signaling whereby: overactivation of WNT/β-catenin promotes stemness, dysregulation of Notch inhibits differentiation, suppression of BMP promotes hyperproliferation, and imbalance of FGF/WNT/BMP/NOTCH enhances cellular plasticity and invasion. Further discussion of emerging therapies targeting epithelial markers and regulatory factors, emphasizing current development in novel, precision-based approaches in CRC treatment is also included.

## 1. Introduction

**Figure 1:**
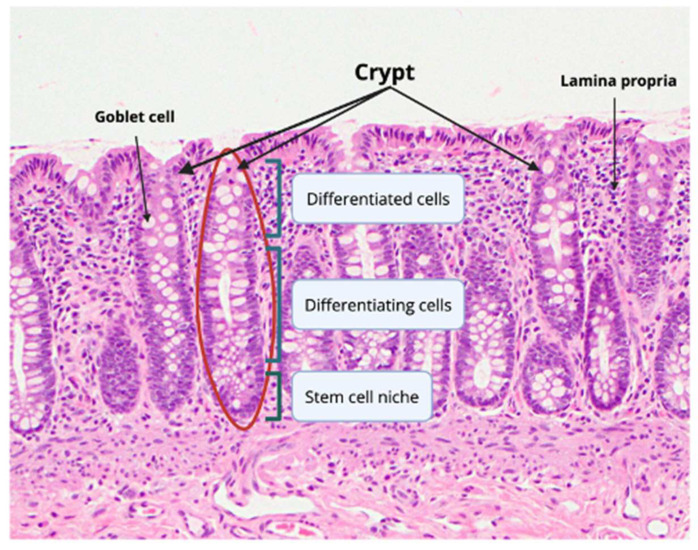
Histology of colon crypts. Colonic crypts are invaginations of the epithelial lining (highlighted by red circles and black arrows). The crypt base (small bracket at the bottom) serves as the stem cell niche, housing stem cells. As cells migrate upward, they begin to differentiate (middle blue bracket), and upon reaching the crypt top (top blue bracket), they become fully differentiated, including goblet cells. The surrounding connective tissue is the lamina propria, which supports the crypt microenvironment. Courtesy of PathologyOutlines.com, contributed by Lizhi Zhang, M.D [5]. Created in Biorender. Nguyen, A.L. (2025) https://BioRender.com/adaugcu.

The gastrointestinal (GI) tract is one of the most anatomically and functionally complex organs in the human body; it is functionally necessary for nutrient absorption, metabolic homeostasis, and defense against infection [1]. The human large intestine (colon) plays a unique role in the GI tract and digestive system. While the small intestine is responsible for most nutrient absorption, the colon mainly functions to form stool from indigestible food and prepare it as waste for excretion from the body [2]. The colon does this by slowly absorbing the remaining water and electrolytes in the food material, which is facilitated by the colonic contractions that move the waste along [2,3]. The colon contents promote the chemical digestion of food material by the presence of a high density of microbes that reside within the colon. The human colon epithelium has a quick turnover capacity, unique structure, and specialized cell types to achieve its physiological function and protect itself from potentially harmful fecal matter and microbes [2]. As the most rapidly self-renewing tissue in the human body, the intestinal lining comprises a monolayer of epithelial cells characterized by their swift tissue turnover capacity [1]. This quick turnover is theorized to be necessary due to the mechanical stress and risk of abrasions from pushing waste along, as well as chemical threats from the high density of microbes and highly concentrated fecal matter [4]. Because of its unique epithelial architecture, the colon can renew every 3–5 days to combat such stressors. Histologically, the colonic epithelium comprises crypts, small tube-like pockets within the intestinal wall (Figure 1). Colonic crypts are made up of specialized intestinal cell types that serve particular functions [2]. The proportions of cell types within the crypt are kept in balance to maintain the cellular hierarchy and tissue organization needed structure and function of the colon. Dysregulation of the colon is associated with multiple gastrointestinal tract diseases including inflammatory bowel disease (IBD) and colorectal cancer (CRC). Many studies have sought to investigate the role that each of these specialized cell types may play in progression of the diseases of the GI tract, particularly CRC, the second leading cause of cancer death in the US, and the concerning rise in early-onset CRC. Single-cell sequencing technology has advanced our understanding of the changes at the cellular level during the adenoma-to-carcinoma sequence. This review seeks to provide a comprehensive overview of each of the cell types in the colon, their function in the colon, and the changes in these epithelial cell populations during CRC. We also provide a review of a select relevant signaling pathways essential in cell fate specification and their changes during CRC. We highlight one of the challenges in treating CRC, tumor heterogeneity and cellular plasticity. A key contributor to this plasticity is the reversible processes of epithelial–mesenchymal transition (EMT) and mesenchymal–epithelial transition (MET), which are associated with differentiation and de-differentiation. In particular, EMT has been gaining attention as a potential target for therapeutic intervention due to its role in CRC. We also review the current clinical trials targeting EMT.

## 2. Normal Colonic Epithelial Cell Composition

### 2.1. Colonic Cells and Transit-Amplifying Cells

**Figure 2:**
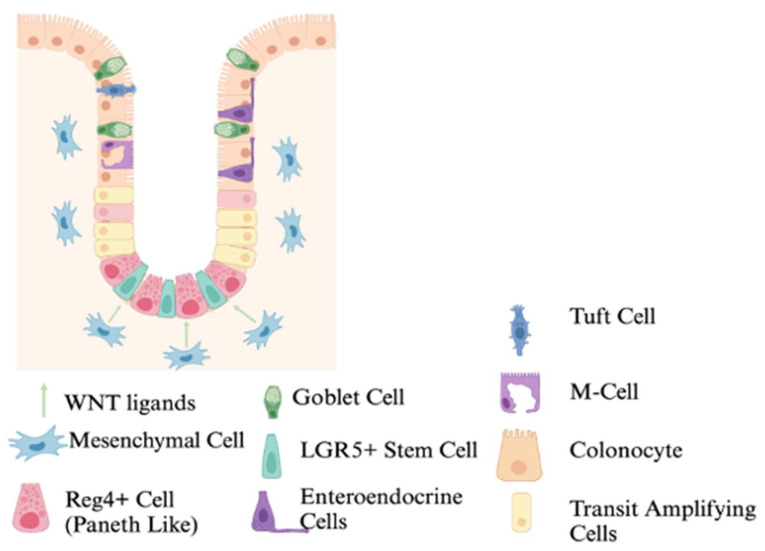
Colonic Crypt Structure. The base of the crypt is the stem cell niche, containing LGR5+ stem cells (progenitors for all other cell types) and Reg4+ Paneth-like cells (provide support and a protective environment for stem cells). These cells divide to produce TA cells (rapidly dividing progenitor cells), which undergo rapid proliferation to maintain cell turnover. As these cells migrate upward, they differentiate into specialized mature cells, including Colonocytes (responsible for water and electrolyte absorption), Goblet cells (secrete protective mucus), Enteroendocrine cells (sense the gut environment and release hormones), Tuft cells (sensory cells involved in immune and inflammatory responses), and M-cells (transport antigens to immune cells). Mesenchymal cells (support the epithelial cells and produce signaling molecules) are also shown in the lamina propria. The upward green arrows indicate the WNT ligands produced by some mesenchymal cells that maintain stemness in the crypt base. Mesenchymal cells that are near the crypt base secrete WNT ligands, which are crucial for maintaining the stem cell population. Mesenchymal cells located further away from the crypt base do not secrete WNT ligands. The mature differentiated cells continue moving upward until they finally undergo apoptosis and are replaced.

At the bottom of the crypt lies the stem cells (SCs) responsible for the self-renewal and regeneration of all the mature cell types (Figure 2). Continuously dividing SCs reside at the bottom of the crypt in a specialized region referred to as the SC niche [2]. These SCs can be identified by their expression of the cell surface marker leucine-rich repeat-containing G protein-coupled receptor 5 (LGR5) [6]. In addition to LGR5, Olfm4, Ascl2, Bmi1, and Smoc2 are recognized markers of active crypt base columnar stem cells [7,8,9], Ascl2 plays a key role in stem cell maintenance, whereas Bmi1 and Smoc2 help distinguish between quiescent and proliferative stem cell populations [7,8,9]. The LGR5+ SCs can differentiate into all the mature intestinal cell lineages [6]. The differentiated mature cells can be divided into two overarching categories based on their function: secretory (colonocyte and M cells) or absorptive (Paneth-like, goblet, enteroendocrine, and tuft cells) [2]. The SCs produce a transit-amplifying (TA) cell population where cells transition into differentiating absorptive and secretory progenitor cell types (Figure 2). The rapid division of TA cells promotes migration of cells along the crypt axis and they undergo additional rounds of cell division as they move up the crypt (Figure 1 and Figure 2) [10]. Ki-67 is a well-established proliferation marker commonly used to identify TA cells [11]. High Ki-67 levels indicate active epithelial renewal, whereas reduced expression reflects impaired repair, and overexpression is associated with crypt hyperplasia or tumorigenesis [11].

### 2.2. Colonocytes and Goblet Cells

As cells move up the crypt, they then differentiate into their mature terminally differentiated cell types. Most cells will form either colonocyte (absorptive) or goblet cells (secretory). These cell types are the most dominant mature cells in each of the two lineages. The crypt comprises about 15% absorptive colonocytes, specializing in water reabsorption, and 20% goblet cells, specializing in mucus production [12]. Histologically, colonic enterocytes, termed colonocytes, are polarized with a distinct basolateral end and apical membrane that contain microvilli that increase the surface area and aid in water absorption [13]. Colonocytes maintain epithelial barrier integrity and regulating oxygen consumption to support anaerobic microbiota, sustaining colon homeostasis [14]. Dysfunction in colonocytes leads to increased luminal oxygenation, imposing an increased susceptibility to inflammation and metabolic disease [14]. Goblet cells produce and secrete mucus that lubricates and protects the surface of the epithelium against bacteria (Figure 1 and Figure 2) [2]. Goblet cell-secreted mucins form the mucus layer, a critical barrier that protects against pathogen invasion and sustains mucosal [15,16]. Several markers are commonly used to identify goblet cells: Atoh1, the key transcription factor driving goblet cell differentiation; Muc2, the predominant mucin protein; and Fcgbp and Clca1, which contribute to mucus network stabilization and hydration [17]. Disruption of goblet cell function or marker expression—such as reduced Muc2 production—compromises this barrier, weakens mucosal defenses, and increases susceptibility to infections and chronic inflammatory diseases, including IBD and CRC [18,19]. A recent comprehensive review of goblet cells is available in [7]. Maintaining crypt homeostasis requires a delicate balance of proliferation, differentiation, apoptosis, and extrusion, and many signaling pathways are essential to the dynamic equilibrium of the colonic epithelium. Homeostasis is also key to maintaining organization of cells in colonic crypts including less-common cell types such as Paneth-like cells, M cells, tuft cells, and enteroendocrine cells.

### 2.3. Paneth-Like Cells

Although the epithelium in colon, unlike small intestine, lacks Paneth cells, the colon does contain a related cell type, deep secretory cells (DCS), that were first identified with regenerating family member 4 (REG4) in the mouse colon [20]. Lysozyme, secretory phospholipase A2 (sPLA2), human defensin 5 (HD5) and MMP7 (matrilysin) have been used as markers for Paneth-like cells while REG4 is a highly specific marker for DCSs, the functional analog of Paneth-like cells in the colon [20,21,22,23,24]. Paneth-like cells make up just under 10% of the colonic epithelium and they are located at the base of the crypt interspersed with the SCs [12]. Paneth-like cells, or Paneth-like cell metaplasia (PCM), are rare in normal colon and typically found at the base of the crypts interspersed with SCs (Figure 2) [25,26,27]. They are morphologically characterized by the columnar to pyramidal shape and the presence of antimicrobial peptides—packed in apical cytoplasmic granules [26,28]. The main function of Paneth-like cells is to regulate the local microbiota and maintain the epithelial barrier by secreting the antimicrobial products into the crypt lumen [25,28,29]. Moreover, they are also referred to as SC bodyguards because they contribute to the maintenance and regeneration of the SC compartment by providing essential niche factors [25,29]. While true Paneth-like cells are a defining feature of the small intestine, the appearance of Paneth-like cells in the colon is generally considered an adaptive change, often associated with pathological conditions such as injury, inflammation, or disrupted signaling pathways [24,25,27,30]. For more information, a recent in-depth review on deep crypt secretory cells can be found in Schumacher et al., 2023 [31].

### 2.4. M Cells

Another rare cell type in the colon crypt is the M cell (Figure 2). M cells, also known as microfold cells, are specialized epithelial cells found in the intestinal tract. These cells play a crucial role in the mucosal immune system by facilitating the uptake and transport of antigens from the lumen to underlying immune cells [32]. In the normal colon, M cells are primarily located in the follicle-associated epithelium overlying lymphoid follicles, such as Peyer’s patches [32,33]. It is important to note that M cells are distinct from Mo cells (also called M cells), which are enteroendocrine cells found in the crypts of the small intestine and secrete motilin [32,34]. M cells in the colon contribute to the maintenance of intestinal homeostasis by sampling luminal contents and presenting antigens to the immune system [35,36]. This process is essential for the development of appropriate immune responses to both commensal and pathogenic microorganisms [32]. The presence and function of M cells in the normal colon highlight the complex interplay between the epithelium and the immune system in maintaining gut health [32]. GP2 and PGLYRP1 are established markers that distinguish M cells, with GP2 being a crucial player in mediating uptake of pathogenic antigens [37,38]. Altered marker expression or a dysregulated M cell population leads to impaired immune tolerance and increased risk to pathogen invasion [39].

### 2.5. Tuft Cells

Tuft cells, also known as brush cells, are a unique type of epithelial cell found in the GI tract, including the colon making up less than 0.5% of intestinal epithelium (Figure 2) [40,41]. These cells are characterized by their distinct morphology, featuring a tuft of microvilli protruding into the lumen [40,42]. They play a crucial role in the innate immune response by acting as sentinels for the detection of parasites and other potential threats [43]. Tuft cells express taste receptors and other chemosensory molecules, allowing them to sense and respond to various luminal stimuli [44,45]. Upon activation, tuft cells release interleukin-25, which stimulates type 2 innate lymphoid cells and initiates a protective immune response [46,47]. In the normal colon, tuft cells are crucial in maintaining homeostasis and initiating appropriate immune responses to potential pathogens [40,46,47]. DCLK1 is the most common and specific marker for tuft cells, and POU2AF2 is another marker denoting additional lineage-specific transcription of tuft cells [40,48]. DCLK1 upregulation is frequently observed in infection or carcinogenic transformation [49].

### 2.6. Enteroendocrine Cells

Enteroendocrine cells (EECs) are specialized epithelial cells of the gastrointestinal tract, including the colon, responsible for sensing luminal contents and secreting a variety of hormones and peptides [50,51]. In the normal colon, EECs account for roughly 1% of the epithelial population and are found as single cells distributed throughout the mucosa; most are located within the SC niche, where they are suggested to play a role in maintaining SC quiescence (Figure 2) [52,53,54]. Synaptophysin (SYP) and chromogranin A (ChgA) are widely used markers of enteroendocrine cells (EECs), as their immunoreactivity distinguishes EECs from other crypt cells. In the colon, reduced numbers of ChgA^+^ cells have been observed in conditions associated with hormone dysregulation, such as IBD, CRC, and type 2 diabetes [48,55]. EEC differentiation occurs in three key stages: (i) the secretory fate is first induced in cells outside the crypt base through absence of Notch signaling, (ii) Neurog3-driven lineage specification then directs these precursors toward one of several hormone-secreting programs, and (iii) finally, hormone plasticity allows EECs to dynamically regulate their hormonal output in response to local signaling gradients along the crypt, so a single EEC can produce different hormones at various times throughout its lifespan [41,56,57]. Functionally, EECs secrete a diverse range of hormones such as glucagon-like peptide-1 (GLP-1), peptide YY (PYY), and serotonin [51]. In the colon, the EEC population is mainly composed of L cells (producing GLP-1, PYY, and oxyntomodulin) and enterochromaffin cells (producing serotonin) [51,58]. These cells regulate important physiological processes, including intestinal motility, secretion, and appetite control [58,59,60]. Beyond this, EECs are crucial sensors of the intestinal microenvironment, detecting microbial metabolites and coordinating host responses to maintain gut homeostasis [60,61]. Notably, recent evidence suggests that EECs have a considerably longer turnover rate than other epithelial cells: while enterocytes are replaced every 4–5 days, serotonin (5-HT)–producing EECs can persist for up to 28 days [62]. These 5-HT+ EECs are found exclusively in the upper half of the crypt within the mucosal fold, an area enriched by visceral sensory fibers [62]. Their high plasticity and prolonged lifespan underscore the diverse and complex roles that EECs play in maintaining healthy colon function and development. A recent review of EECs can be found here [50,51].

## 3. Disruption of Colonic Epithelium in CRC

In the healthy colon, a tightly regulated hierarchy of epithelial cell types-including LGR5+ SCs, TA progenitors, absorptive colonocytes, goblet cells, EECs, tuft cells, and rare M cells-ensures the continual renewal and functional specialization of the mucosa. This homeostatic balance is orchestrated by a network of transcription factors (e.g., *CDX2*, *ATOH1*, *NEUROG3*) and signaling pathways, which direct progenitor cells toward specific lineages while maintaining SC identity at the crypt base [63,64,65,66,67]. However, during tumorigenesis, this system becomes profoundly disrupted, resulting in the emergence of aberrant cellular states and enhanced plasticity that drive tumor progression, heterogeneity, therapy resistance, and loss of tissue organization.

One of the earliest and most significant changes observed during CRC initiation is the reactivation of fetal-like or injury-responsive gene expression programs in epithelial cells [68]. Single-cell RNA sequencing along with patient-derived organoids studies have demonstrated that both primary tumors and metastatic lesions are enriched for cells expressing fetal markers such as LGR5, ASCL2, and PROX1, which are typically restricted to embryonic development or tissue repair [69,70]. These cells often display reduced expression of differentiation markers (e.g., CDX1, VIL1), enabling them to evade lineage constraints and acquire stem-like properties [69,70,71]. The plasticity of LGR5+ cancer stem cells (CSCs) is particularly notable; these cells can dynamically transition between proliferative, invasive, and quiescent states in response to microenvironmental cues, such as WNT ligands, inflammatory cytokines, and hypoxia [72,73,74]. Importantly, even differentiated colonocytes and goblet cells can undergo dedifferentiation, re-entering the cell cycle and acquiring stem-like or mesenchymal features through processes such as EMT [75,76]. This section will focus on recent findings on the changes in each of the colonic cell types in CRC.

### 3.1. Stem Cells During Tumor Progression

**Figure 3:**
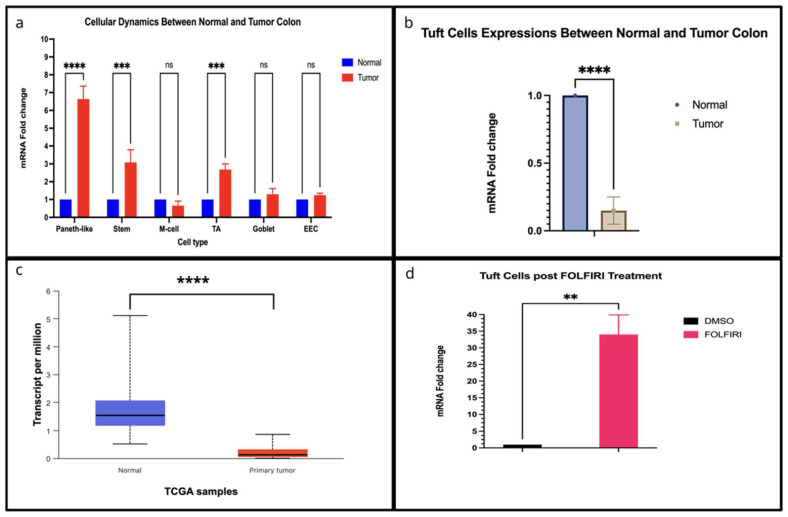
Change in cellular distributions in CRC. (**a**) Change in the expressions of different cell types between normal and tumor tissues from Broad cohort [85]. Molecular markers used for each cell type are indicated in Table 1. (**b**) Change in the expressions of tuft cells between normal and tumor tissues from Broad cohort [85]. (**c**) Change in the expressions of tuft cells between normal and tumor tissues from TCGA [86]. (**d**) Change in the expressions of tuft cells after FOLFIRI treatment versus Control (DMSO) from Mzoughi et al. [87]. ns = Not significant, **: *p* < 0.005, ***: *p* < 0.0005, ****: *p* < 0.0001.

During CRC progression, colonic LGR5+ SCs undergo profound changes that underpin tumor growth, heterogeneity, and metastatic potential [77]. In the normal colonic crypt, LGR5+ SCs reside at the crypt base and serve as the principal source for epithelial renewal [78]. As CRC develops, LGR5 expression is significantly upregulated in tumor tissues compared to normal mucosa, and LGR5+ CSCs exhibit enhanced WNT/β-catenin signaling, clonogenicity, and tumor-initiating capacity (Figure 3a) [79,80,81,82] LGR5+ cells display unique mechanical properties: they are stiffer, adhere more strongly to the extracellular matrix, move more slowly, and show higher nuclear YAP activity than their LGR5 counterparts, traits that confer survival advantages during metastatic colonization [83,84].

**Table 1:** Molecular markers of cell types in the colonic crypt (* indicates markers used for expression analyses).

Cell Type	Marker	References
Mesenchymal cells	*CD34*, *gp38* (podoplanin), αSMA–/low	[63]
Paneth-like cells	*Lyz* *, *CD24*, *MMP7*, *REG4*	[20,21]
Goblet cells	*Atoh1*, *Fcgbp*, *Clca1*, *Muc2* *	[17]
Stem cells	*Lgr5* *, *Ascl2*, *Smoc2*, *Olfm4*, *Bmi1*	[8,9,20]
Enteroendocrine cells	*SYP* *, *ChgA*	[48]
Tuft cells	*DCKL1* *; *POU2AF2*	[40,48]
M cells	*GP2* *, *PGLYRP1*	[37,38]
Transit Amplifying cells	*MKI67* * (Ki-67)	[11]

However, the SC compartment in CRC is highly plastic. LGR5+ and LGR5− populations can interconvert, with LGR5− cells capable of reacquiring LGR5 expression under specific conditions, such as after drug withdrawal or environmental changes [84]. This dynamic plasticity allows tumors to adapt to selective pressures and may contribute to drug resistance, as LGR5+ cells can convert to a drug-resistant LGR5− state during chemotherapy, only to regain LGR5 expression and proliferative potential once the stress is removed [87,88,89]. Notably, LGR5 expression tends to increase during the adenoma–carcinoma sequence but declines at invasive tumor fronts and in budding cancer cells, suggesting context-dependent regulation during tumor progression and invasion [87].

Beyond LGR5+ cells, evidence from lineage tracing shows that slower-dividing, radioresistant LGR5− KRT19+ stem-like populations outside the crypt base can also serve as a reservoir for tumor initiation, further highlighting SC plasticity in CRC [90]. The functional significance of LGR5 in CRC remains complex, with some studies associating high LGR5 expression with poor prognosis and others linking it to better clinical outcomes, possibly reflecting differences in tumor stage, microenvironment, or methodological approaches [91].

Overall, the SC compartment in CRC is characterized by increased abundance, enhanced plasticity, and altered mechanical and molecular properties [70]. These changes enable tumors to sustain growth, adapt to environmental and therapeutic pressures, and facilitate metastatic dissemination, underscoring the central role of SC dynamics in CRC progression [92,93]

### 3.2. Transit Amplifying Cells During Tumor Progression

During CRC progression, TA cells normally situated just above the SC compartment are responsible for rapid proliferation that reflects the broader dysregulation of epithelial hierarchy [94]. In healthy tissue, TA cells act as a proliferative bridge between slowly cycling SCs and terminally differentiated colonocytes, goblet cells, and other specialized cell types [95]. However, as colorectal tumors develop, TA cells increasingly exhibit altered proliferative dynamics and disrupted differentiation trajectories [96,97]. Enhanced WNT/β-catenin signaling, a hallmark of CRC, drives excessive proliferation of both stem and TA cells, leading to an expanded pool of undifferentiated or partially differentiated progenitors [98,99].

Single-cell analyses have revealed that, along the continuum from normal mucosa to adenoma and carcinoma, there is a progressive increase in cells with stem-like and TA-like features, while the proportion of fully differentiated cells declines (Figure 3a) [100]. Epigenetic and transcriptional reprogramming in TA cells that occur during tumor progression includes increased accessibility of chromatin regions associated with WNT target genes (such as those containing Lymphoid Enhancer Factor-1 (LEF1) and T-cell Factor (TCF) motifs), and decreased accessibility of regions linked to differentiation-promoting factors like KLF and HOX family transcription factors [101,102,103]. This shift supports a “progenitor state” in which TA cells may fail to complete terminal differentiation, thereby fueling tumor growth and heterogeneity [101,102,103].

Moreover, the proliferative output of TA cells in CRC is further amplified by mutations in key signaling pathways-including MAPK/ERK, PI3K/AKT, and Notch, which collectively disrupt the normal balance between proliferation and differentiation [97,104,105,106]. These molecular changes not only expand the TA cell compartment but also enable these cells to contribute directly to the malignant epithelial population, sometimes bypassing normal lineage restrictions [97,104,105,106]. Overall, TA cells in CRC are characterized by enhanced proliferation, impaired differentiation, and increased plasticity, playing a pivotal role in the expansion of undifferentiated tumor cell pools and the progression of colorectal neoplasia.

### 3.3. Goblet Cells During Tumor Progression

Recent studies have significantly advanced our understanding of how goblet cells change during CRC progression, revealing nuanced patterns of differentiation loss and molecular regulation. Although the single cell-RNA sequencing data does not indicate any significant change in the entire CRC cohort, goblet cells have been found to be more associated with a distinct category of CRC: mucinous CRC (mCRC) (Figure 3a) [107]. Goblet cells, which are responsible for producing the mucus barrier of the colon through the secretion of MUC2 and TFF3, are progressively altered in mCRC [107]. Comprehensive profiling of nearly 80 CRC-derived cell lines and direct tumor specimens has led to the classification of CRCs into five distinct subgroups based on MUC2 and TFF3 expression, ranging from nearly normal goblet cell differentiation to complete absence [107]. Notably, about 30% of CRCs exhibit a previously unrecognized phenotype characterized by high TFF3 but absent MUC2 expression, suggesting the existence of a unique subgroup with partial goblet cell features [107,108]. This subgroup may have important implications for tumor biology, as TFF3 is known not only for its role in mucus stabilization but also for promoting epithelial restitution and potentially stimulating tumor growth [109,110]. The loss of goblet cell identity in CRC is increasingly attributed to epigenetic mechanisms, particularly changes in DNA methylation, rather than direct genetic mutations [107,111,112,113,114]. Key transcription factors and regulators-including ATOH1, SPDEF, CDX1, CDX2, GATA6, and notably LGR5, are implicated in the selection against goblet cell differentiation, with LGR5 expression being inversely correlated with goblet cell features and potentially functioning as a regulator of differentiation rather than simply a marker of stemness [107,111,112,113,114]. Single-cell and trajectory analyses further demonstrate that, while some mucinous adenocarcinoma cancer cells retain goblet cell-like gene expression profiles early in tumorigenesis, there is a marked reduction in canonical goblet cell markers such as MUC2, TFF3, SPINK4, and REG4 as tumors progress [115,116,117,118]. This loss of differentiation is a hallmark of aggressive, poorly prognostic CRC subtypes and it is considered to be a key evolutionary step favoring uncontrolled growth over specialized function [107]. Overall, these recent findings underscore that goblet cell loss in CRC is a dynamic and heterogeneous process, driven primarily by epigenetic reprogramming and selection against differentiation. This evolving landscape not only refines our understanding of CRC biology but also opens new avenues for diagnostic stratification and potential therapeutic targeting of specific CRC subgroups based on goblet cell differentiation status.

### 3.4. Paneth-Like Cells During Tumor Progression

Recent research has revealed that Paneth-like cells undergo striking changes during CRC progression, playing dynamic roles in tumor development and the tumor microenvironment [25]. In early colorectal adenomas, Paneth-like cells accumulate in regions with heightened WNT/β-catenin and Notch signaling, where they provide niche factors that support SC maintenance and proliferation [119,120]. Their presence in adenomas is associated with increased vessel density, elevated expression of SC markers such as EphB2, and poorer disease-free survival, underscoring their contribution to early tumorigenesis and SC niche formation [119,121].

Mechanistic studies using lineage tracing and genetic models have demonstrated that Paneth-like cells can act as cells of origin for intestinal tumors, particularly in the context of inflammation and loss of tumor suppressors such as *APC* [122,123,124]. When *APC* is ablated specifically in Paneth-like cells, especially under inflammatory conditions, these cells can initiate adenoma formation and, with additional mutations in *KRAS* or *TP53*, drive increased tumor multiplicity and progression to dysplasia and invasive cancer [122,123,124]. Single-cell RNA sequencing and organoid models have further shown that OLFM4+ SCs in advanced CRC can give rise to Paneth-like cells, especially when Notch signaling is inhibited [25]. These Paneth-like cells, identified by markers such as DEFA6 and LYZ, are essential for propagating tumor organoids and are found in tumor regions where cell adhesion is lost, suggesting they play a key role in maintaining the CSC niche and supporting tumor growth [25,29,125,126,127].

Additionally, recent single-cell analyses have identified distinct populations of LYZ+ cancer cells with Paneth-like cell characteristics throughout different stages of CRC progression [128]. These cells are enriched for pathways involved in protein secretion and glycolysis, processes central to Paneth-like cell function, and their emergence is dependent on DKK2 signaling, which is crucial for generating the CSC niche and facilitating metastatic outgrowth [129]. The presence of Paneth-like cells in CRC has also been linked to immune modulation, potentially influencing T-cell infiltration and the tumor immune microenvironment [130,131].

Collectively, these new findings from the past three years highlight that Paneth-like and Paneth-like cells, through their plasticity and niche-supporting functions, are increasingly recognized as key contributors to colorectal tumor progression, SC maintenance, and possibly immune regulation, making them a potential target for novel therapeutic strategies

### 3.5. M Cells and Tuft Cells During Tumor Progression

Recent research has begun to clarify the roles and dynamics of both M cells and tuft cells during CRC progression, highlighting their contributions to tumor heterogeneity and the evolving tumor microenvironment. M cells, which are specialized for antigen sampling in gut-associated lymphoid tissue and rare in the normal colon, remain poorly characterized in CRC [32,132]. However, emerging evidence suggests that their presence may be increased in tumors, particularly in the context of chronic inflammation or lymphoid aggregates, potentially influencing immune surveillance and tumor-immune interactions [36]. Although direct mechanistic studies are limited, the plasticity of the epithelial compartment in CRC suggests that non-canonical cell states, including M cell-like phenotypes, may arise as part of the broader dedifferentiation and lineage reprogramming observed during tumor progression [70]. This is supported by single-cell analyses showing that primary tumors and metastases can give rise to a spectrum of non-canonical epithelial states, some of which may include rare or ectopic cell types [70].

Tuft cells, in contrast, have been the focus of several recent studies that underscore their expanding and potentially tumor-promoting role in CRC. They, marked by DCLK1 and POU2AF2, are rare chemosensory epithelial cells involved in tissue repair and inflammation [45,133,134]. There are some controversies about Tuft cells’ expression in primary CRC tumors. There are studies using mouse models and on human CRCs that show tuft cell abundance is significantly increased compared to normal tissue, and their expansion is associated with tumor progression [135,136,137]. However, our expression analyses using single-cell RNA sequencing from 35 pairs of normal tumors from the Broad cohort and RNA sequencing from The Cancer Genome Atlas (TCGA) show that tuft cells have lower expression in tumors [85,86]. Interestingly, using the RNA sequencing from Mzoughi et al. [87], we found that tuft cells are dramatically upregulated upon FOLFIRI treatment, a chemotherapy regimen composed of folinic acid (leucovorin), fluorouracil (5-FU), and irinotecan, commonly used to treat advanced or metastatic CRC, suggesting their role is dominantly in treatment response (Figure 3d). Notably, recent genetic studies have linked variation at chromosome 11q23.1 to both increased CRC risk and reduced tuft cell abundance, implicating genes such as POU2AF2 in mediating tumor-suppressive effects [138]. These findings indicate that while tuft cell expansion is suggested to promote tumor growth, specific genetic contexts may alter their abundance and functional impact.

Together, these new insights reveal that both M cells and tuft cells undergo dynamic changes during CRC progression, with tuft cells in particular emerging as active players in tumor development and microenvironmental remodeling. Their abundance, molecular signatures, and functional roles are now recognized as important facets of CRC biology and may provide novel biomarkers or therapeutic targets as our understanding deepens.

### 3.6. EECs During Tumor Progression

Although the expression analysis from the Broad cohort indicates no significant change in EECs population between normal and tumor (Figure 3a), recent studies have highlighted the complex and stage-specific contribution of EECs to tumor progression in CRC. In early-stage CRC, hyperactivation of the WNT/β-catenin pathway suppresses neuroendocrine differentiation and disrupts the hormonal regulation that normally maintains epithelial homeostasis, leading to the reduction in the EEC populations [139,140]. Moreover, this depletion is suggested to create a permissive environment for tumor initiation due to the low abundance of growth-inhibitory hormones, such as somatostatin [139,141]. Notably, serotonin (5-HT), a hormone produced by EECs, is upregulated in the serum of CRC patients [142]. 5-HT upregulation can promote CRC tumorigenesis via activation of RhoA/ROCK/YAP signaling [142]. Moreover, depletion of EECs leads to diminished GLP-1 secretion, potentially resulting in immunosuppressive macrophage polarization and impaired cytotoxic T-cell activity [143,144,145]. Moreover, ectopic expression of hormones like ghrelin, another hormone produced by EECs in tumors has been found to correlate with metastatic potential in cancers [146,147,148]. Together, these findings highlight EECs as dynamic players in CRC pathogenesis, potentially in both early tumor development and during tumor progression.

## 4. Regulatory Signaling in Normal and Neoplastic Colonic Crypts

The renewal and differentiation of colonic epithelial cells are coordinated by several key pathways, including WNT, Notch, bone morphogenetic protein (BMP), and fibroblast growth factor (FGF) signaling. WNT signaling is highly active at the crypt base, where it drives self-renewal and crypt expansion by sustaining the proliferation of stem and TA cells [149]. Similarly, Notch signaling is activated in the stem cell zone to regulate cell fate, either preserving progenitors in an undifferentiated state or promoting their differentiation toward the absorptive lineage) [150,151]. FGF signaling also contributes by supporting stem cell proliferation and pluripotency, thereby maintaining crypt architecture [152]. In contrast, BMP signaling becomes more active as cells migrate upward along the crypt, counteracting WNT and Notch activity to promote maturation into specialized cell types [153,154]. Disruption of the balance among these pathways can result in aberrant proliferation, impaired differentiation, and defective epithelial renewal, ultimately predisposing to neoplasia or inflammatory disease. Thus, the coordinated interplay of WNT, Notch, BMP, and FGF signaling is essential for maintaining colonic epithelial homeostasis. The following sections will explore in greater detail the role of each pathway in normal colon physiology and their dysregulation in CRC.

### 4.1. WNT Signaling in Normal Colon

Many signaling pathways are important in maintaining the homeostasis of the colon, and WNT signaling ranks among the most important pathways. The WNT signaling pathway is critical in maintaining stemness in the colon’s SC niche and crypt homeostasis. The activity of WNT is highest in the SC niche and is a significant regulator of SC renewal and differentiation. WNT signaling progressively decreases up the crypt axis [2]. The WNT signaling is driven via the activity of the receptor complex of Frizzled and LRP5/6 when bound by WNT ligands. WNT ligands are glycoproteins secreted from cells after modification in their endoplasmic reticulum [155]. The mechanism of how WNT is transferred to the target cell has yet to be elucidated. The colon, unlike the small intestine, does not receive WNT signals from any epithelial cell types in the crypt, but recent research has shown colon cells receive WNT signals from the surrounding mesenchyme or stroma [10,156,157]. Once bound to the receptor complex on the target cell, WNT ligands lead to the recruitment of the destruction complex to the cell membrane, which leads to an increase in available β-catenin. Stabilized β-catenin can now translocate to the nucleus. Once in the nucleus, it induces changes in transcription by binding to transcription factors LEF1 and TCF [158]. This causes a conformational change that displaces Groucho, a transcriptional repressor that allows for β-catenin binding and its mediated transcription. Key WNT target genes driven by β-catenin-mediated transcription include *c-MYC*, *LGR5*, *cyclin D1*, *c-JUN*, *OCT4*, *SOX2* among others [159,160,161,162]. However, when the WNT ligand is absent, the destruction complex of APC, AXIN, disheveled, CK1A, and GSK3β will form. Notably, APC is a scaffold protein that binds to β-catenin and the other scaffold protein AXIN, which interacts with GSK3β, CK1A, and disheveled [158]. CK1A phosphorylates β-catenin at a serine residue, allowing GSK3β to phosphorylate β-catenin even further. These two-phosphorylation events cause β-catenin to be recognized by the ubiquitin ligase b-TrCP, which ubiquitinates phosphorylated β-catenin. This ubiquitination marks it for proteasomal degradation [163]. WNT is considered a short-range signal produced by the colonic mesenchyme, and a WNT gradient is established as the amount of WNT decreases through cell division and cellular movement away from the SC niche [164,165]. The SCs in the colonic crypt are characterized by their high WNT activity [4,12,166]. Several key experiments both in vitro and in vivo have shown the necessity of the WNT signaling pathway for SC maintenance as demonstrated by knocking out key WNT proteins that leads to a loss of SCs, and that the overactivation of this pathway increases the number of LGR5+ SCs [2,10,125,167,168,169,170,171]. Thus, WNT signaling is essential in maintaining stemness and the balance of proliferation and differentiation driven by colonic SCs.

### 4.2. WNT in CRC

**Figure 4:**
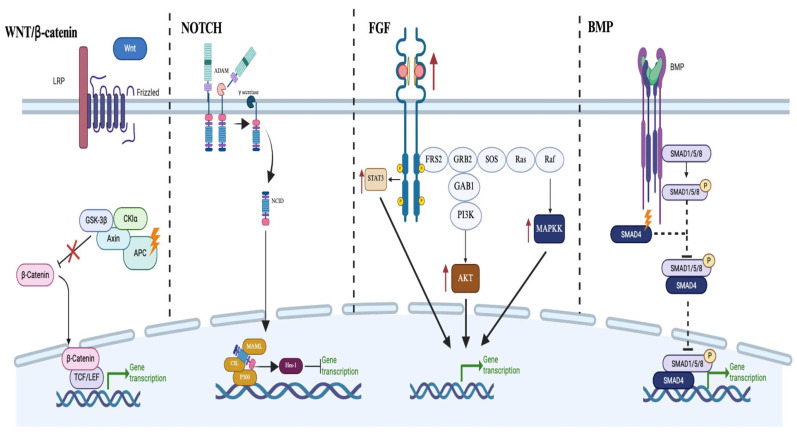
Dysregulations of major signaling pathways in CRC. WNT/β-catenin pathway: In a healthy state, the β-catenin destruction complex (composed of APC, GSK3β, CK1α, and Axin) tags β-catenin for degradation. However, in CRC, APC mutations impair this complex, leading to the accumulation of β-catenin. This accumulated β-catenin translocates to the nucleus and binds to TCF/LEF transcription factors, activating the transcription of genes that promote cell proliferation and survival. APC: adenomatous polyposis coli; GSK3β: glycogen synthase kinase 3 beta; CK1α: casein kinase 1 alpha; TCF/LEF: T-cell factor/lymphoid enhancer factor. NOTCH pathway: Ligand binding to the NOTCH receptor triggers a series of proteolytic cleavages, releasing the Notch intracellular domain (NICD). The NICD then moves into the nucleus, where it binds with CSL and MAML to activate the transcription of target genes, such as *HES1.* This activation represses genes necessary for differentiation, contributing to the loss of differentiation characteristic of CRC. NICD: Notch intracellular domain; CSL: CBF1/Su(H)/Lag-1; MAML: mastermind-like protein. FGF pathway: The binding of FGF to its receptor, FGFR, leads to the hyperactivation of two key downstream cascades: the PI3K/AKT pathway and the Ras/MAPK pathway. This is mediated by adaptor proteins like FRS2, GRB2, and GAB1. The resulting increase in transcription of pro-survival and pro-proliferative genes contributes to uncontrolled cell growth. FGF: fibroblast growth factor; FGFR: FGF receptor; FRS2: FGFR substrate 2; GRB2: growth factor receptor-bound protein 2; SOS: Son of Sevenless; GAB1: GRB2-associated-binding protein 1; PI3K: phosphoinositide 3-kinase; AKT: protein kinase B; MAPK: mitogen-activated protein kinase. BMP pathway: The binding of BMP to its receptor induces the phosphorylation of SMAD1/5/8. These phosphorylated proteins then form a complex with SMAD4 and translocate into the nucleus to regulate gene expression. A loss of function in SMAD4 is a common event in CRC, which disrupts this signaling and leads to the loss of transcription of tumor suppressor genes. BMP: bone morphogenetic protein; SMAD1/5/8: receptor-regulated SMADs; SMAD4: common-mediator SMAD. Created in BioRender. Nguyen, A.L. (2025) https://BioRender.com/k9shegg.

The constantly renewing epithelial barrier that makes up the colonic lining also puts the colon at greater risk for cancer than other slower self-renewing tissues. The risk of cancer is correlated with tissues that require a large amount of SC divisions to maintain homeostasis due to the additional risk of random mutation accumulation [172]. CRC follows a stereotype progression from normal epithelium to carcinoma with the stepwise acquisition of well-characterized genetic mutations referred to as the adenoma to carcinoma sequence [173]. The ‘first hit’ is an activating mutation in the WNT signaling pathway, with 93% of all CRC containing an activating mutation in this pathway–particularly in *APC* (Figure 4) [2,174]. It is also hypothesized that CRC-initiating events occur in the SC niche because mutated cells must compete with healthy SCs for limited niche space; otherwise, they would be pushed out of the crypt and shed into the intestinal lumen within 3–5 days due to crypt turnover [2,175,176]. A recent single-cell analysis was performed on healthy colon, precancerous adenomas, and CRC; the authors showed a progressive increase in WNT signaling throughout carcinoma progression as well as an increase in SCs at each step of the carcinoma progression coupled with a decrease in differentiated cell types [100]. Becker et al. found that SCs increased in adenomas to 25% and showed a significant decrease in colonocyte progenitors, colonocytes, and a depletion of immature and mature goblet cells [100]. Another study quantified SCs by performing in situ hybridization to detect *LGR5* mRNA on normal colonic crypts and CRC [177]. Normal crypts showed that 6% of cells were LGR5+ SCs, while in CRC, the number increased by ten-fold in some cases [177]. This subpopulation of cells that display stemness-like features in malignant tumor populations is known as CSCs. CSCs are believed to be drivers of cancer growth and have been linked to the initiation and progression of CRC as well as therapeutic resistance and recurrence [178]. CRC CSCs have stem-like properties similar to normal colonic SCs and embryonic SCs, including unlimited division and multi-lineage differentiation ability. One of the properties of CRC CSCs is constitutive activation of WNT and other signaling pathways that affect proliferation, differentiation, and drug resistance. The WNT signaling pathway supports the maintenance of pluripotency in embryonic SCs, and CSCs re-express genes that typically are highly expressed in embryonic SCs, including *OCT4*, *SOX2*, and *c-MYC*. Pluripotency is defined as the ability of cells to differentiate into all mature cell populations in the human body [179]. *OCT4*, *SOX2*, and *c-MYC* are required to maintain CSCs in an undifferentiated state [180]. These pluripotency factors are usually expressed during embryogenesis and become re-expressed during cancer development, which is why they are frequently referred to as oncofetal drivers [181,182,183]. These pathways and expression changes in pluripotent genes allow CSCs to possess self-renewal properties that enhance tumorigenicity and recurrence after chemotherapy.

### 4.3. Fibroblast Growth Factor Signaling in Normal Colon

The Fibroblast Growth Factor (FGF) signaling pathway plays a crucial role in regulating colonic crypt development and homeostasis [184,185]. This complex molecular cascade involves multiple components and interactions that ultimately influence cell proliferation and survival in colonic epithelium [184,185]. FGF signaling in the normal colon is characterized by spatial gradients of several FGF ligands, especially FGF1, FGF2, FGF7, FGF9, and FGF10 [186,187,188,189,190]. Gradients of FGF are established within the colonic crypts, where higher concentrations are near the crypt base and while lower concentrations toward the differentiated region of the crypt, thus defining a boundary between proliferating and differentiating cells [187,188,191,192,193].

At the core of this pathway is the FGF receptor (FGFR). The FGF ligands induce dimerization in the FGFR through the help of co-factors, then the receptor subsequentially becomes activated, and this activation initiates a signaling cascade that involves several downstream effectors [185].

One of the key downstream pathways activated by FGF signaling is the β-catenin/TCF-4 signaling axis [194,195]. Indeed, FGF signaling has been shown to maintain high levels of β-catenin mRNA expression and sustain activation of the TCF4/β-catenin pathway [194,195]. The β-catenin/TCF-4 complex acts as a transcriptional activator, inducing gene expression in cell proliferation and stemness [185,194,195,196].

FGF signaling regulates the number of epithelial SCs and influences Paneth-like cell differentiation through the MAPK cascade, particularly the ERK1/2 and p38 MAPK pathways [185,196]. This dual role highlights the importance of FGF signaling in maintaining the delicate balance between SC self-renewal and differentiation in the intestinal crypt. In summary, the FGF signaling pathway coordinates a complex molecular network that regulates intestinal crypt development and maintenance. By modulating the WNT/β-catenin and MAPK pathways, and interacting with other key developmental pathways, FGF signaling ensures proper SC function, proliferation, and differentiation in the colonic epithelium.

### 4.4. FGF Signaling in CRC

FGF signaling plays a crucial role in CRC development, progression, and metastasis [185]. The FGF family consists of numerous ligands and receptors that contribute to various aspects of tumor biology, with FGF19 and its receptor FGFR4 emerging as key players in CRC pathogenesis [185].

FGF19, an endocrine FGF primarily involved in bile acid homeostasis under normal conditions, is often aberrantly elevated in CRC, contributing to tumor growth and metastasis [185,197,198]. This overexpression is strongly associated with poor prognosis, advanced disease stages, and liver metastasis in CRC patients [197,198]. Mechanistically, FGF19 promotes EMT, a critical process for tumor invasion and dissemination, through FGFR4-mediated activation of key signaling pathways [197,199].

The molecular pathways activated by FGF19-FGFR4 signaling are complex and interconnected (Figure 4). Upon ligand binding, FGFR4 activates multiple downstream cascades, including the GSK3β/β-catenin, ERK1/2, and AKT pathways [185,197,198,199]. These pathways collectively enhance cell proliferation, migration, invasion, and resistance to apoptosis [199]. The GSK3β/β-catenin axis is particularly important in CRC, as it is a key component of the WNT signaling pathway, which is frequently dysregulated in colorectal tumors.

In addition to these canonical pathways, FGF19-FGFR4 signaling also activates the JAK2-STAT3 axis, particularly in the context of CRC liver metastases [197].

The complexity of FGF19-FGFR4 signaling encompasses its ability to drive oncogenic transcriptional programs through intermediates such as ELF4 [198]. FGF19 upregulates ELF4 expression via the ERK1/2-SP1 axis, which subsequently activates downstream targets like SRC and FGFR4 itself, creating a positive feedback loop that amplifies the metastatic potential of CRC cells [198].

The complex interplay between different FGF ligands and receptors in CRC extends beyond the FGF19-FGFR4 axis. For instance, FGF9 is overexpressed in colorectal tumor tissues compared to normal tissues, with higher expression levels associated with advanced-stage disease and chemotherapy resistance [200]. Similarly, the FGFR3-IIIc splice variant shows increased expression in advanced tumors, enhancing proliferation and survival in CRC models [198,201].

The multifaceted nature of FGF signaling in CRC makes it an attractive target for therapeutic interventions. More specifically, targeting the FGF19-FGFR4 axis with selective inhibitors, such as fisogatinib, have demonstrated efficacy in suppressing tumor progression and metastasis [198].

### 4.5. Notch Signaling in Normal Colon

The NOTCH signaling pathway plays a crucial role in normal colon development and homeostasis, particularly in regulating cell fate decisions and maintaining the balance between proliferation and differentiation [202,203]. In the normal colon, NOTCH signaling is active in the SC niche and TA compartment of the crypt [78]. NOTCH receptors (NOTCH1-4) are activated by ligands (Delta-like and Jagged) expressed on adjacent cells, leading to proteolytic cleavage and release of the NOTCH intracellular domain (NICD) [204,205,206]. The NICD translocate to the nucleus, forming a complex with RBPJ and MAML1 to activate target gene transcription [204,205,206]. Key NOTCH target genes include *HES1*, which represses the expression of *ATOH1*, a master regulator of secretory cell lineage differentiation [204,205,206]. This mechanism promotes the absorptive cell fate (colonocytes) while inhibiting secretory cell differentiation (goblet cells, enteroendocrine cells, and tuft cells) [207]. NOTCH signaling also interacts with other pathways, such as WNT, to regulate SC maintenance and differentiation [208].

### 4.6. Notch Signaling in CRC

In CRC, NOTCH signaling is frequently dysregulated, contributing to tumor initiation, progression, and metastasis [97,104]. Aberrant activation of NOTCH signaling occurs in a significant proportion of CRC cases, often due to overexpression of NOTCH receptors or ligands Figure 4 [97,104,209]. This hyperactivation leads to increased proliferation and reduced differentiation in tumor cells. NOTCH signaling also promotes EMT, enhancing tumor cell invasion and metastasis [209]. The crosstalk between NOTCH and other oncogenic pathways, such as WNT and KRAS, further amplifies its tumorigenic effects. For instance, NOTCH can synergize with WNT signaling to maintain CSCs and promote their self-renewal [210]. Given its critical role in CRC pathogenesis, the NOTCH pathway has emerged as a promising therapeutic target. Several NOTCH inhibitors, including γ-secretase inhibitors and monoclonal antibodies targeting NOTCH receptors or ligands, are currently being evaluated in preclinical and clinical studies for CRC treatment [97,104,211].

### 4.7. Bone Morphogenetic Protein Signaling in Normal Colon

The Bone Morphogenetic Protein (BMP) pathway plays a crucial role in normal colon development and homeostasis. In the normal colon, BMP signaling acts as a key regulator of SC activity and differentiation along the crypt axis [191]. BMP ligands, primarily produced by mesenchymal cells, bind to type I and type II serine/threonine kinase receptors on the cell surface [212]. This binding triggers the phosphorylation of SMAD1/5/8 proteins, which then form a complex with SMAD4 and translocate to the nucleus to regulate gene expression [213]. BMP signaling is highest at the top of the crypt and decreases towards the base, creating a gradient that is inverse to that of WNT signaling [214,215]. This gradient is maintained by BMP antagonists such as Noggin and Gremlin, which are secreted by mesenchymal cells at the base of the crypt [214,215]. The BMP pathway also regulates crypt fission and intestinal epithelial cell migration, making it one of the most crucial pathways in maintaining homeostasis in the colonic epithelium [216].

### 4.8. BMP Signaling in CRC

In CRC, the BMP pathway is frequently dysregulated, contributing to tumor initiation and progression. Mutations in BMP pathway components, particularly SMAD4, are common in CRC and are associated with poor prognosis [217]. Loss of SMAD4 leads to reduced BMP signaling, resulting in increased SC self-renewal and impaired differentiation (Figure 4) [217]. This shift in cellular composition contributes to the expansion of the SC pool observed in CRC. BMP pathway alterations also impact other oncogenic pathways, such as WNT, to promote tumor growth and metastasis. For instance, a loss of BMP signaling can enhance WNT pathway activation, further driving SC proliferation and tumor formation [218]. In advanced CRC, some tumors may exhibit a paradoxical increase in BMP ligand expression, which has been associated with enhanced metastatic potential [217,218]. Understanding these molecular mechanisms has led to exploring BMP pathway modulation as a potential therapeutic strategy in CRC, with both BMP agonists and antagonists showing promise in preclinical studies [217,218,219].

### 4.9. EMT in CRC

The process of EMT is associated with CRC progression and metastasis. EMT is the reversible processes of EMT and mesenchymal–epithelial transition (MET), which are associated with differentiation and de-differentiation [220,221]. EMT has been shown to be closely related to the progression of CRC and key step for progression to advanced stage cancer. EMT can be triggered by many of the signaling pathways discussed above. EMT is a highly conserved process involving morphological changes where cells lose polarity, break intercellular junctions, and transform from immobile epithelial cells into mobile mesenchymal cells. Key molecular hallmarks of EMT progression are a downregulation of epithelial markers like E-cadherin and upregulation of mesenchmyal markers like N-cadherin and Vimetin [221,222]. This enhanced mobility promotes invasion and metastasis. EMT is also linked to the acquisition of stem-like properties in both normal and cancerous cells and linked to treatment resistance [222]. EMT has been linked to treatment resistance to conventional treatments like chemotherapy and radiotherapy as well as evidence for resistance to immunotherapy [223]. For an in-depth, recent review of EMT in CRC, see Tan et al. (2022), Lu et al. (2023) and Nie et al. (2025) [220,221,222]. Moreover, to make studying EMT even more nuanced, tumors contain a heterogeneous population of cells exhibiting various degrees of EMT, and even cells with partial EMT have been shown to possess metastatic potential [224].

## 5. Cellular Plasticity and EMT-Targeted Therapies in CRC

### 5.1. Cellular Plasticity in CRC

**Figure 5:**
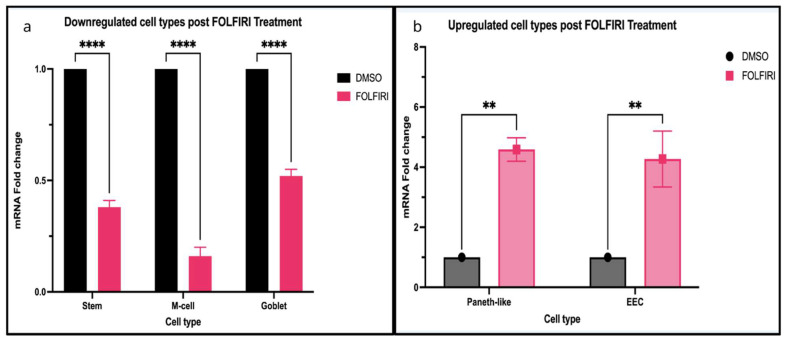
Phenotypic changes upon FOLFIRI treatment. (**a**) mRNA fold-changes in downregulated cell types (Stem, M-cell, Goblet) post-FOLFIRI compared to DMSO (**b**) mRNA fold change in upregulated cell types (Paneth-like, EEC) post-FOLFIRI. Data are presented as mean ± SEM. Statistical significance: **: *p* < 0.01, ****: *p* < 0.0001. DMSO: Dimethyl sulfoxide; FOLFIRI: 5-FU, leucovorin, and irinotecan; EEC: Enteroendocrine cell.

Phenotypic changes in CRC following chemotherapy serves as a hallmark of cellular plasticity, underlying the tumor’s ability to adapt and survive standard treatments [225]. In the metastatic stage, cancerous colon cells may lose their original colonic identity and reprogram to express other intestinal cell types, such as esophagus, stomach, small intestine [87]. Recent findings indicate the oncofetal reprogramming in CRC allows the LGR5+ SC population to re-express fetal-like gene to sustain the hybrid state, potentially evade FOLFIRI treatment [87]. In fact, upon analyzing the RNA sequencing results in Mzhoughi et al. [87], we found distinct phenotypic changes in CRC model post FOLFIRI treatment. Namely, LGR5+ SCs, M cells, and goblet cells are significantly downregulated, while Paneth-like cells, EECs, and tuft cells are significantly upregulated, with tuft cells showing the most dramatic change (Figure 3d and Figure 5a,b). Not only limited to chemotherapy response, recent studies also suggested that the EECs play a crucial role in promoting treatment resistance through its secretory function as they secrete pro-survival factors within the tumor microenvironment [226]. A central regulator of EEC specification is the STAT3 pathway [227]. Recent studies show that lysine-specific demethylase 1A (LSD1) and REST corepressor 2 mediate STAT3 demethylation and chromatin binding that interferes with EEC specialization [227]. LSD1 plays a critical role in EEC progenitor formation suggesting that EEC-mediated resistance potentially occurs through epigenetic reprogramming [225,228,229]. These findings highlight the dynamics and high plasticity of tumor cells, which enables them to adapt to combination drug treatments. Also, the tumor microenvironment and loss of lineage-restricting factors further drive these transitions, allowing cancer cells to adapt to new niches and evade therapies [225,229].

### 5.2. EMT-Targeted Therapies in CRC

Thus far, this paper has detailed the epithelial cells that comprise the colonic epithelial cell populations, the changes these populations undergo during CRC progression, and a review of select signaling pathways. One of the many challenges in treating CRC is the tumor heterogeneity and plasticity that are present. The preceding section highlighted how this plasticity can affect the effectiveness of standard treatments. A key contributor to this plasticity is the reversible processes of EMT and mesenchymal–epithelial transition (MET), which are associated with differentiation and de-differentiation [220,221]. EMT has been shown to be closely related to the progression of CRC and key step for progression to advanced stage cancer. EMT can be triggered by many of the signaling pathways discussed above. In particular, EMT has been gaining attention as a potential target for therapeutic intervention due to its role in CRC [221,222,223].

EMT represents a promising target for preventing primary tumors from becoming aggressive, reducing the risk of metastasis and recurrence after tumor resection. However, the adaptability and complexity of the signaling pathways involved in tumorigenesis make it difficult to develop drugs that specifically target EMT and many seek to try to target it indirectly. Despite this, several companies have initiated Phase I and II clinical trials. Early trials focused on detecting and measuring EMT-associated transcription factors. For instance, trial NCT04323813 utilizes circulating tumor cells to assess disease progression, metastasis, and recurrence risk in CRC (Table 2). Other trials examine EMT states in blood and tumor tissues to explore combinations of EMT-targeted therapy with immunotherapy or conventional chemotherapy to improve patient outcomes [220,224,230].

Currently, two promising EMT-related targets being explored in trials for solid tumors are clusterin and netrin-1 [231,232,233,234]. Clusterin is a glycoprotein implicated in inducing EMT through the TGF-β, PI3K/Akt, and NF-κB pathways [234]. Netrin-1, on the other hand, is a secreted laminin-related protein that has recently been suggested as an EMT regulator through the MAPK/ERK and TGF-β signaling cascades [235,236]. Targeting clusterin or netrin-1 may inhibit these oncogenic pathways, thereby suppressing EMT and enhancing sensitivity to chemotherapies [236,237]. However, since both proteins also play roles in tissue homeostasis, neuronal guidance, and cell survival, systemic inhibition may cause adverse effects, highlighting the need for extensive research to balance efficacy and safety in clinical applications.

**Table 2:** Clinical trials targeting EMT in CRC and other solid tumors.

Sponsor and Clinical Trial Gov ID	Title of Study	Status of Clinical Trial	Summary	Stage
Istituto Clinico Humanitas NCT04323813	High Levels of EMT-TFs for the Diagnosis of CRC	Unknown	An observational and diagnostic study that measures mRNA levels of genes involved in EMT in peripheral blood samples of CRC patients and healthy controls, to determine the presence of disease, its progression and risk of recurrence.	Observational and Diagnostic Study
Alethia Biotherapeutics NCT02412462	Phase I Dose Escalation Study of AB-16B5 in Subjects with an Advanced Solid Malignancy	Completed	A clinical study to investigate the safety, pharmacokinetics and pharmacodynamics of AB-16B5 in patients with an advanced solid malignancy. AB-16B5 is a humanized monoclonal antibody that inhibits the activity of the secreted form of clusterin. Eligible subjects will have a disease that has been refractory to prior therapy and is unlikely to benefit from known therapies. No results posted at time of publication.	A Phase I Dose-Escalation Study to Evaluate the Safety, Tolerability and Pharmacokinetics of AB-16B5 in Subjects with an Advanced Solid Malignancy
Alethia Biotherapeutics NCT06225843	Sotevtamab (AB-16B5) Combined With FOLFOX as Neoadjuvant Treatment Prior to Resection of CRC Liver Metastasis (EGIA-003)	Recruiting	This study will recruit 17 CRC patients with liver-dominant metastases. All recruited patients will receive Sotevtamab at a dose of 800 mg once weekly for 6 cycles combined with FOLFOX once every 2 weeks for the first 4 cycles followed by liver metastases resection surgery with or without primary cancer resection. AB-165 (Sotevtamab) a fully humanized monoclonal antibody of IgG2 isotype against tumor-associated secreted clusterin.	A Proof-of-Concept Phase II Trial to Evaluate the EMT Inhibitor Sotevtamab Combined with FOLFOX Administered as Neoadjuvant Treatment Prior to Resection of CRC Liver Metastasis.
Centre Leon Berard NCT05605496	NP137 Clinical and Biological Activities Assessment in Patients With Advanced/Metastatic Solid Tumors Treated by Standard Anti PD-1/PD-L1 Immunotherapies (IMMUNONET)	Recruiting	This study aims to assess the clinical and biological impact of NP137 when added to standard PD-1/PD-L1 blockade with various sensitivity to anti-PD-1/PD-L1 NP137 is an antibody that inhibits netrin-1. NP137 has shown the ability to inhibit EMT. (Xia et al., 2024 [232]) (Cassier et al., 2023 [238])	This study is an open-label, proof-of-concept study assessing the impact of NP137 when added to standard PD-1/PD-L1 blockade therapy of advanced or metastatic solid tumors with various sensitivity to anti-PD-1/PD-L1
National Cancer Institute (NCI) NCT03030417	Indenoisoquinoline LMP744 in Adults With Relapsed Solid Tumors and Lymphomas	Completed	LMP744 (NSC 706744) an inhibitor of topoisomerase 1 damages DNA. This causes cell death. Researchers want to see if it can treat certain kinds of cancer. Exploratory Objective: Evaluate the effect of LMP744 on markers of DNA damage (yH2AX, pNbs1, pATR, ERCC1, RAD51, Topo1cc, Top1, SLFN11) and EMT in circulating tumor cells and pre- and post-treatment tumor biopsies in patients in the expansion cohort.	A Phase I Study to establish the safety, tolerability and the maximum tolerated dose of LMP744 in patients with refractory solid tumors and lymphomas.

We performed a search for such trials using ClinicalTrials.gov with the terms “CRC,” “colon cancer,” and “solid tumor,” along with the term “epithelial–mesenchymal transition” or “EMT.” We identified five relevant studies, listed in the table below. Additional clinical trials that indirectly target EMT are reviewed by Zhang et al. (2021) [224]. These EMT therapies target can work in various ways either: targeting upstream regulators, EMT transcription factors, or targeting mesenchymal cells. We focused our search on clinical trials that directly mention EMT [221].

### 5.3. Future for EMT-Targeted Therapies

EMT is a highly complex molecular process that involves numerous signaling pathways and because of its complexity EMT targeted therapies alone are unlikely to represent a successful clinical strategy due to the many redundancies in the molecular process. However, EMT regulation has shown promise in regulating both drug resistance and metastatic potential. These make EMT targeted therapeutics a strong candidate to be used as an adjuvant with conventional treatments like chemotherapeutic regimens and immunotherapies [221]. Also, due to the inherent heterogeneity of CRC tumors, a combination treatment may be better able to effectively target cancer cells in these dynamic populations.

Although EMT treatments show promise there are many challenges for future clinical usage including the potential for unintended consequences. A treatment that promotes cells to enter a MET could potentially cause cells that have escaped the already primary tumor site to enhance seeding potential of metastatic sites [221,239]. There is also evidence that MET cell states enhance cellular proliferation which could fuel tumor growth [239]. There is also potential for serious side effects on normal cells as EMT is essential in wound healing and could impair this process [223,239]. Many hypothesize that EMT-targeted therapies will need to be administered during an appropriate time in disease progression to be effective and not promote metastasis. This personalized treatment and appropriate timing will need further research to understand the full implications of an EMT targeted treatment on tumors. EMT biomarkers in the future may prove vital for more personalized medicine and more accurate prognostic and diagnostic applications. However, developing these biomarkers has proved challenging due to the inherent plasticity of EMT. A recent review has highlighted the potential markers and challenges in identifying them [223].

## 6. Conclusions

The colon plays an invaluable role in the human GI tract that is made possible by unique epithelial cell populations. Remarkably, this dynamic tissue regenerates itself every 3–5 days while maintaining the balance of its epithelial cell populations. The colonic crypt is kept in homeostatic balance through the various signaling pathways discussed herein. When homeostatic balance is disrupted in colonic epithelia, one outcome that can occur is carcinogenesis. CRC is one of the most common cancers worldwide. However, treatment options for advanced stage CRC are not curative and for stage IV patients the five-year survival is only 12% [240]. Plasticity and tumor heterogeneity are believed to contribute to ineffective treatment options for CRC. The epithelial cell populations experience a profound shift during the adenoma–carcinoma sequence with a decreased in some differentiated cell types and increase in SC- and TA-like cells. These changes in epithelial cell population led to increased plasticity likely contributes to ineffective treatment options A key contributing factor to both plasticity and heterogeneity in CRC is EMT. Indeed, EMT has been linked to treatment resistance, metastasis, and SC characteristics. Current clinical trials for interventions that target EMT in CRC are summarized in Table 2. Although our scientific understanding of EMT has expanded in recent years there is much we have left to uncover about the complex process of EMT and its potential as a therapeutic target. Additionally, a greater understanding of the roles epithelial cell changes play in CRC progression and treatment resistance may help lead to more effective treatment options. There is much left to uncover regarding colonic epithelial cellular populations and their changes during CRC progression and how to translate these findings into effective therapeutics.

## 7. Data Synthesis and Analysis

### 7.1. Gene Expression Analysis from TCGA-COAD

Transcript per million (TPM) values of selected molecular markers (indicated by * in Table 1) were extracted from The Cancer Genome Atlas–Colon Adenocarcinoma (TCGA-COAD) dataset. Expression levels between normal and tumor samples were compared using an unpaired Student’s *t*-test. Significance was set at *** *p* < 0.0001.

### 7.2. Single-Cell Expression Analysis from the Human Colon Cancer Atlas

Mean scaled expression values of selected markers (*) were obtained from the Human Colon Cancer Atlas via the Broad Institute’s Single Cell Portal, comprising 35 matched pairs of normal and tumor tissues. Fold changes were calculated by normalizing tumor expression to the corresponding normal mean. Statistical significance was determined using two-way ANOVA (** *p* = 0.0001; *** *p* < 0.0001).

### 7.3. RNA-Seq Expression Analysis from Mzoughi et al., 2025 [87]

Bulk RNA-seq data from Mzoughi et al., 2025 [87], were used to assess mRNA expression levels of markers listed in Table 1. Differences between Control (DMSO) and FOLFIRI-treated samples were analyzed using Student’s *t*-test (*p* = 0.005).

## Data Availability

No new data were created or analyzed in this study.

## Abbreviations

SC	Stem cell
CRC	Colorectal cancer
FGF	Fibroblast growth factor
BMP	Bone Morphogenetic Protein
CSCs	Cancer stem cells
EMT	Epithelial—Mesenchymal Transition
MET	Mesenchymal—Epithelial Transition
TA	Transit-amplifying
GI	Gastrointestinal
LGR5	leucine-rich repeat-containing G protein-coupled receptor 5
REG4	regenerating family member 4
sPLA2	secretory phospholipase A2
HD5	human defensin 5
PCM	Paneth-like cell metaplasia
GLP-1	glucagon-like peptide-1
PYY	peptide YY
EECs	Enteroendocrine cells
LEF1	Lymphoid Enhancer Factor-1
TCF	T-cell Factor
mCRC	mucinous CRC
TCGA	The Cancer Genome Atlas
FGFR	FGF receptor
NICD	NOTCH intracellular domain
LSD1	lysine-specific demethylase 1A
IBD	inflammatory bowel disease
